# Supplementary material for: Self-assembled Möbius strips with controlled helicity
Source: Nat Commun. 2020 Nov 20;11:5910. doi: 10.1038/s41467-020-19683-z (PMC7680134; doi:10.1038/s41467-020-19683-z)
Supplement: Supplementary file 1 — Supplementary Information [file 41467_2020_19683_MOESM1_ESM.pdf]

Supplementary Information for

Self-assembled Möbius Strips with Controlled Helicity

1. Supplementary figures.

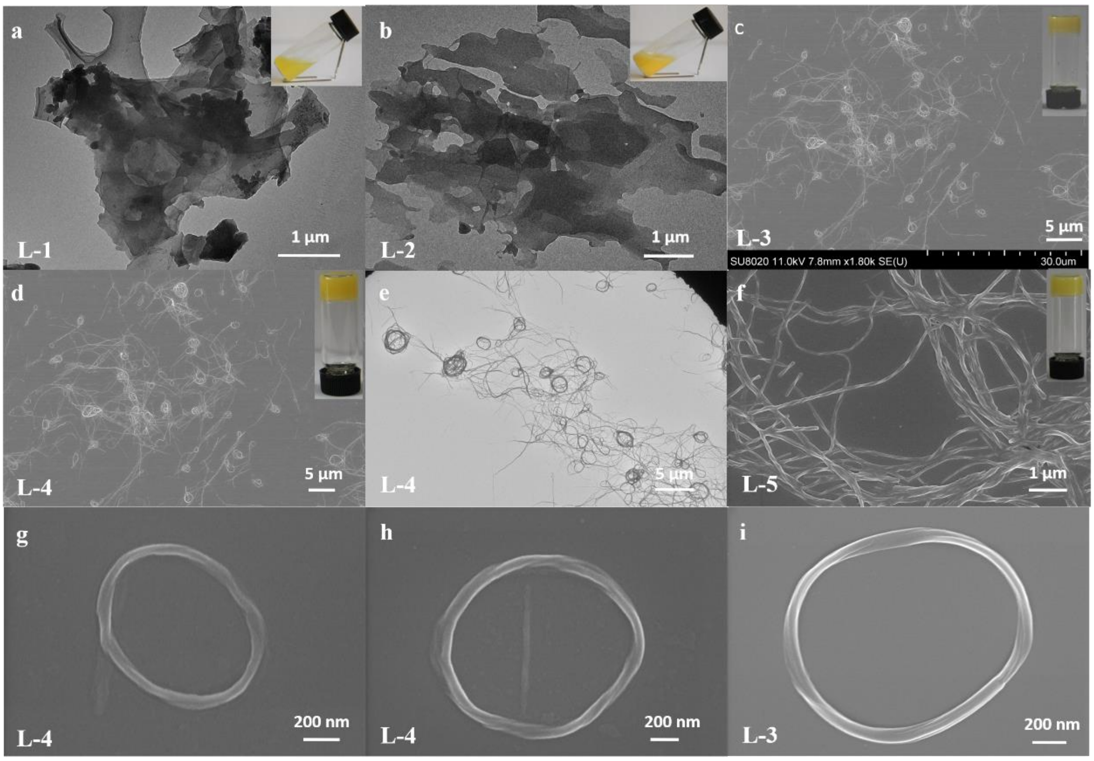

Supplementary Figure 1. SEM and TEM images of self-assemblies of different amphiphiles in water (3.00 mg/mL, pH = 2). **a**, TEM images of L-**1** self-assemblies. **b**, TEM images of L-**2** self-assemblies. Both of them could only form suspension. **c**, SEM images of L-**3** hydrogel. **d**-**e**, SEM and TEM images of L-**4** hydrogel, respectively. **f**, SEM image of L-**5** self-assemblies. **g**-**h**, Nano-toroidal structures observed from L-**4** hydrogel. **i**, A SEM image of M-helical nanotoroid observed from self-assemblies of L-**3**. Notes: the inserted pictures at upper right corner were the photographs of hydrogel vials.

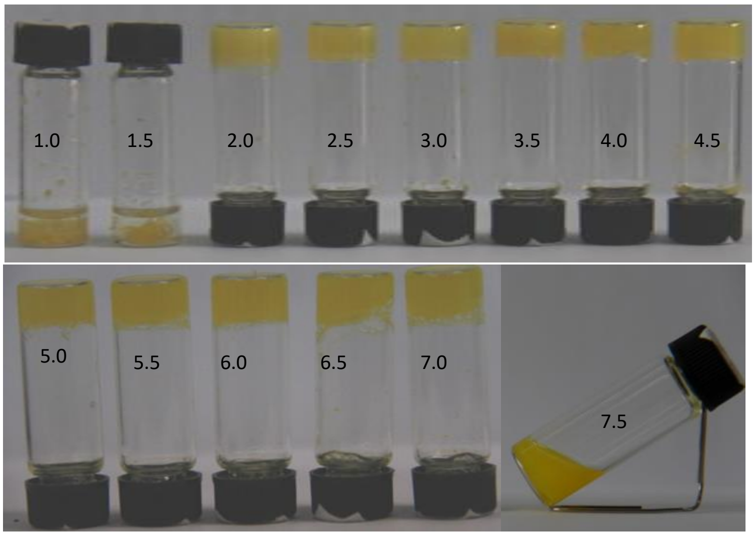

Supplementary Figure 2. Images of precipitates, hydrogels and gelling fluid of L-**3** amphiphile at different pH values, hydrogels were formed when the pH values varied between 2.0 to 7.0, [L-**3**] = 3 mg/mL. The numbers on vials are the pH values.

At lower pH values (1.0 and 1.5), the amphiphile L-**3** could not be dissolved in water even heating to boiling. After cooling to room temperature, only precipitates were obtained. When increasing the pH values from 2.0 to 7.0, L-**3** could be totally dissolved in water after heating to 90 ^{o}C for 30 min, after the transparent solution cooling to room temperature, orange hydrogels could be obtained. Further increasing the pH value to 7.5 or higher values led to the formation of gelling fluid after the same heating-cooling procedures.

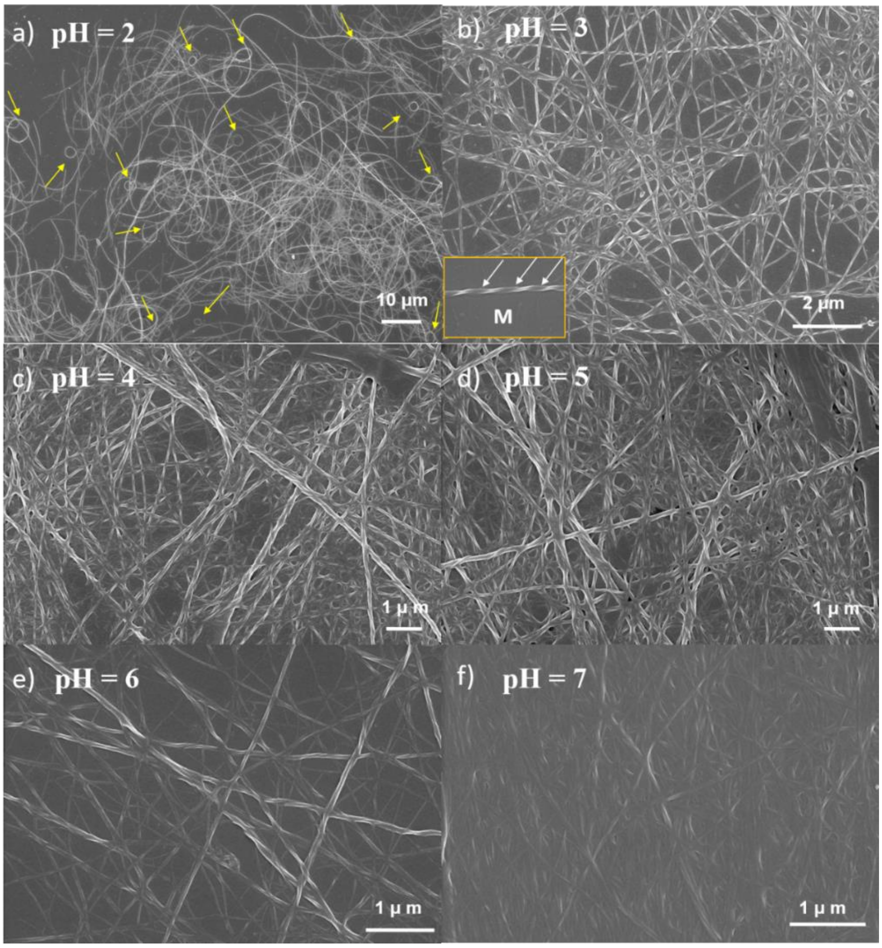

Supplementary Figure 3. SEM images of L-**3** hydrogels at different pH values (the concentration of L-**3** is 3.00 mg/mL). **a**, bended twists and toroids fibers at pH 2.0. Linear twisted fibers at **b**, pH 3.0. **c**, pH 4.0. **d**, pH 5.0. **e**, pH 6.0. **f**, pH 7.0.

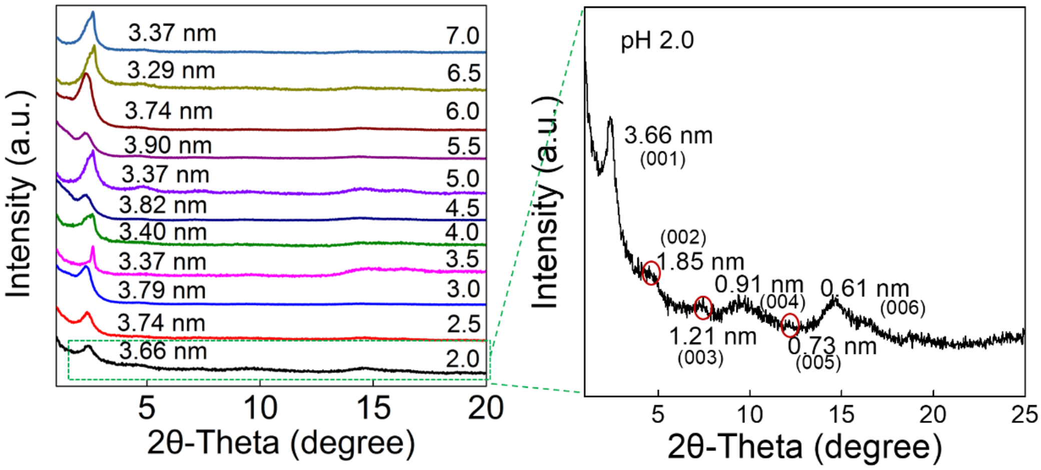

Supplementary Figure 4. XRD patterns of L-**3** xerogels at different pH values. The layer distance 3.66, 1.85, 1.21, 0.91, 0.73 and 0.61 nm of L-**3** at pH 2.0, indicates a well-defined lamellar structure with a d-spacing of 3.66 nm.

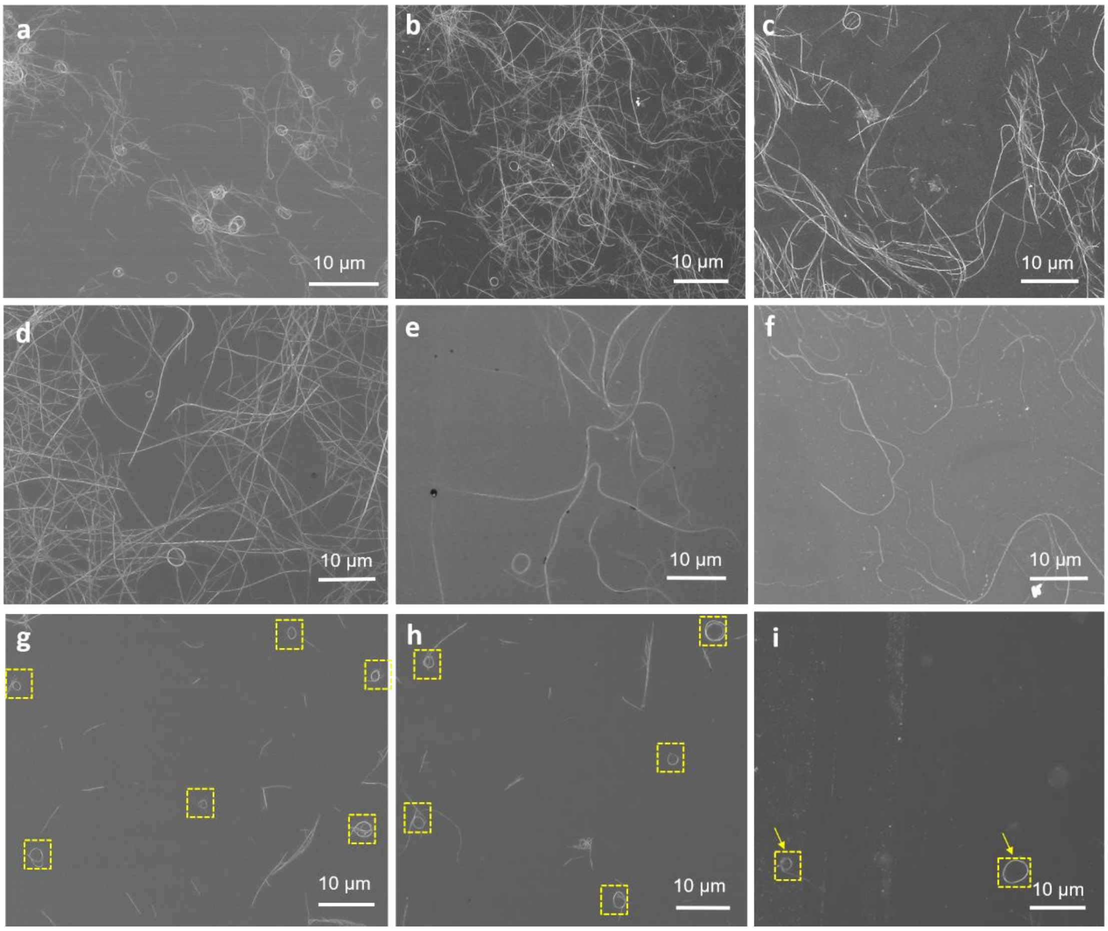

Supplementary Figure 5. SEM images of L-**3** self-assemblies at different concentrations (pH = 2.0). **a,** 3.00 mg/mL (6.80 mM). **b,** 2.00 mg/mL (4.53 mM). **c,** 1.00 mg/mL (2.27 mM). **d,** 0.50 mg/mL (1.13 mM). **e,** 0.20 mg/mL (0.45 mM). **f,** 0.10 mg/mL (0.23 mM). **g, h,** 0.05 mg/mL (0.11 mM). **i,** 0.02 mg/mL (0.05 mM). Samples S5**a-d** can form hydrogels, while samples S5**e-i** form floccules in water. The yellow dashed square and solid arrows in **g-i** are toroidal fibers.

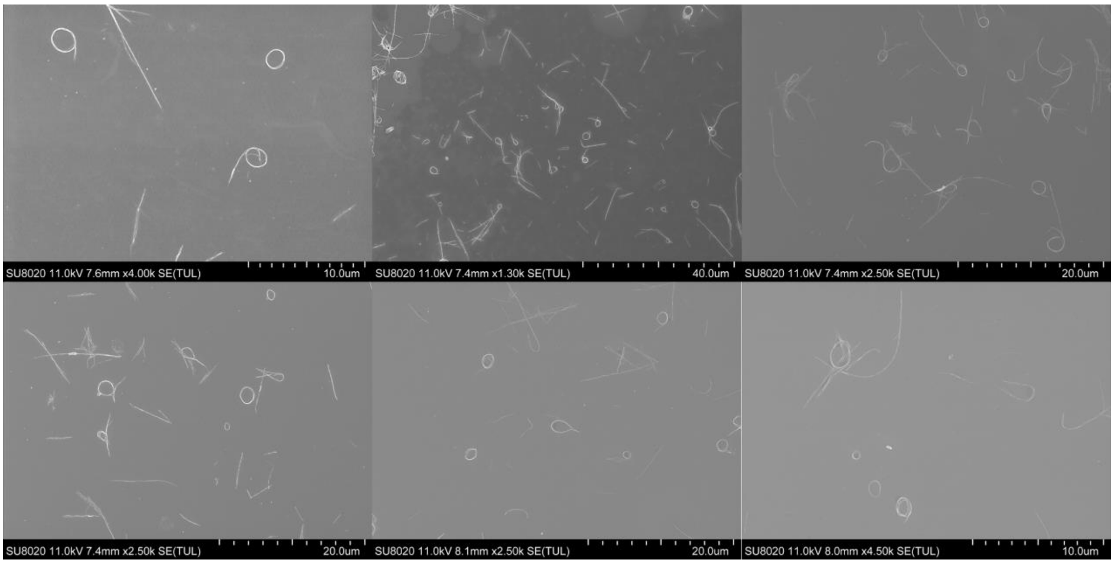

Supplementary Figure 6. SEM images of L-**3** self-assemblies on larger scale (10-40 um) at low concentrations (0.11 mM, pH = 2). Scar bars are marked at the bottom right corner.

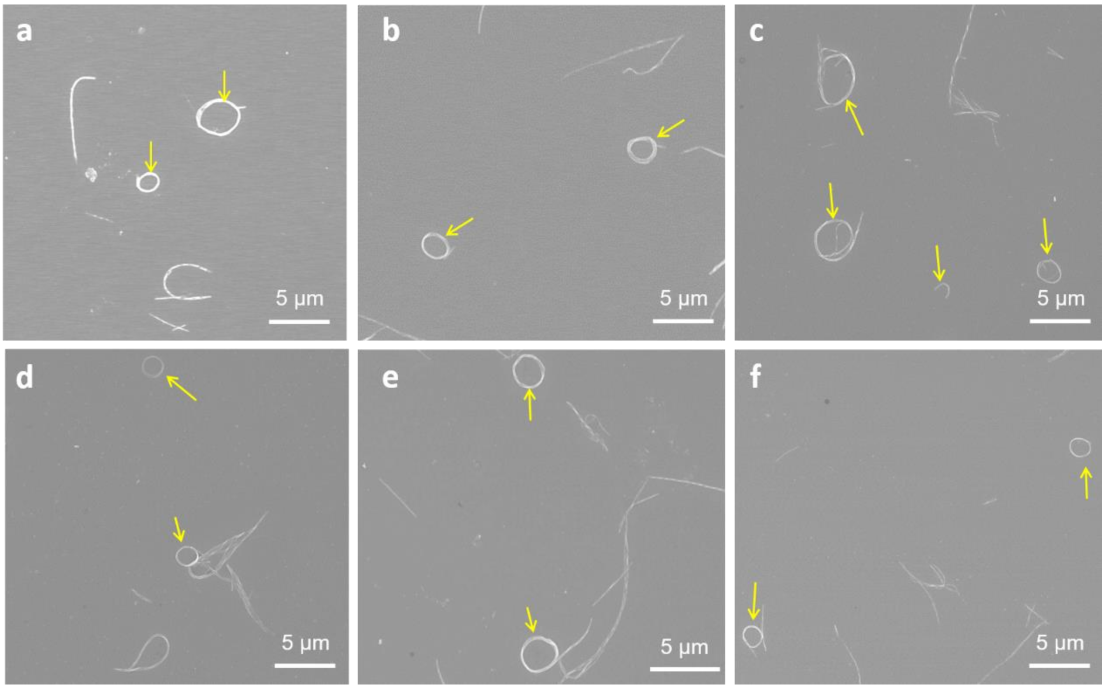

Supplementary Figure 7. SEM images of L-**3** self-assemblies on a smaller scale (5 um) at low concentrations (0.11 mM, pH = 2). The yellow arrows indicate nano-toroids.

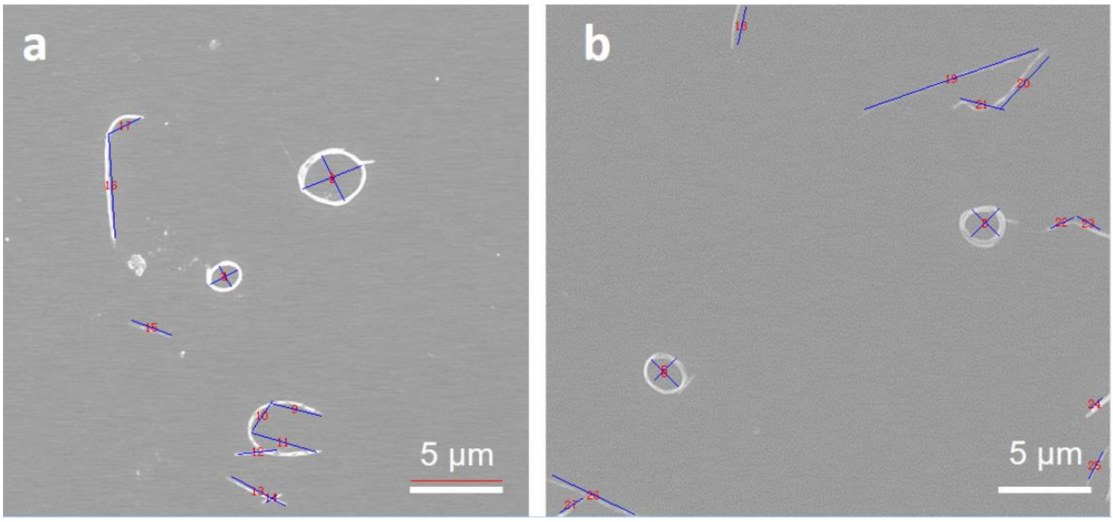

Supplementary Figure 8. SEM images of L-**3** assemblies (0.11 mM, pH = 2). The diameter of toroidal fibers and approximate length of uncyclized fibers were measured to estimate the ratio of circumference of chiral toroidal structures to the length of whole nanofibers**.** Notes: the length of fibers are measured with a software (Nano measurer, version 1.2).

The total fiber length of toroidal structures is 56 μm.

The approximate total fiber length of uncyclized structures is 55 μm.

Therefore, the approximate ratio of toroidal fibers to overall fibers is 56/(56+55) ~ 50 %.

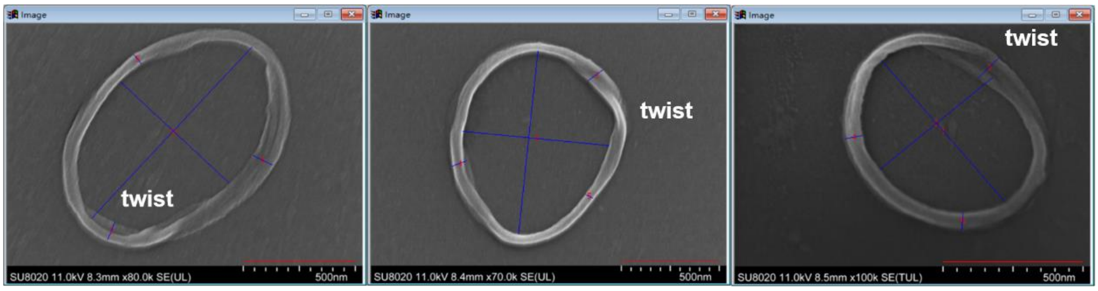

2R = (998 + 684)/2 = 841 nm
2w = (113+103+53)/3 = 90 nm

2R = (958 + 747)/2 = 853 nm
2w = (93+79+44)/3 = 72 nm

2R = (765 + 526)/2 = 646 nm
2w = (105+63+64)/3 = 78 nm

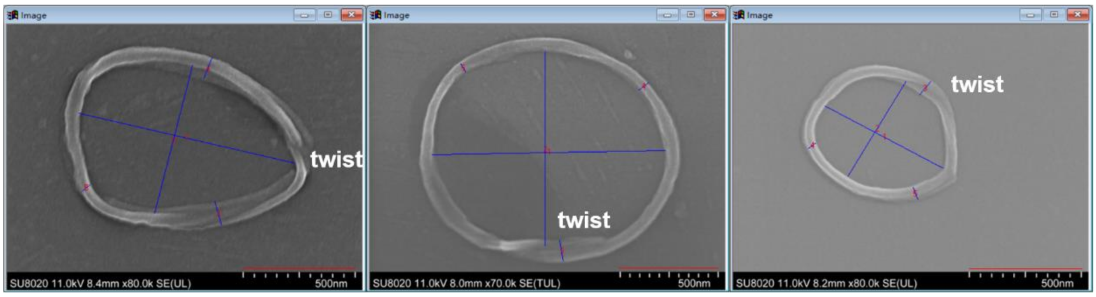

2R = (998.40 + 683.61)/2 = 841.00 nm
2w = (112.62+102.83+52.62)/3 = 89.357 nm

2R = (1178.51 + 981.55)/2 = 1080.03 nm
2w = (110.07+65.10+53.47)/3 = 76.21 nm

2R = (593.09 + 493.88)/2 = 543.49 nm
2w = (80.64+41.76+59.38)/3 = 60.59 nm

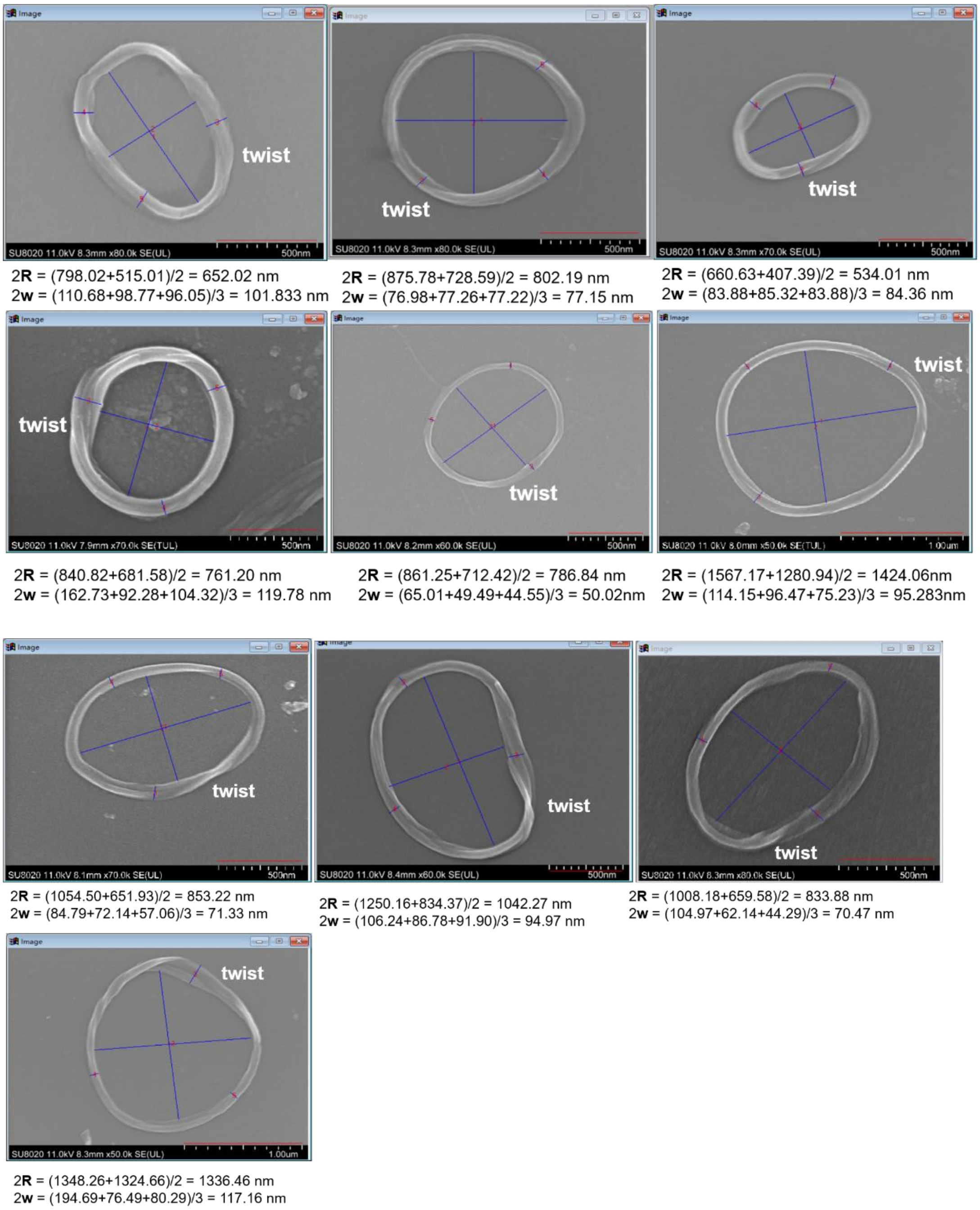

Supplementary Figure 9. SEM images of L-**3** self-assemblies showed chiral toroidal structures with one twist, [L-**3**] = 11 mM, pH =2. 2**R** = (**a**+**b**)/2, **a**: long diameter, **b**: short diameter, 2**w**: average fiber wideness. Scar bars are marked at the bottom right corner.

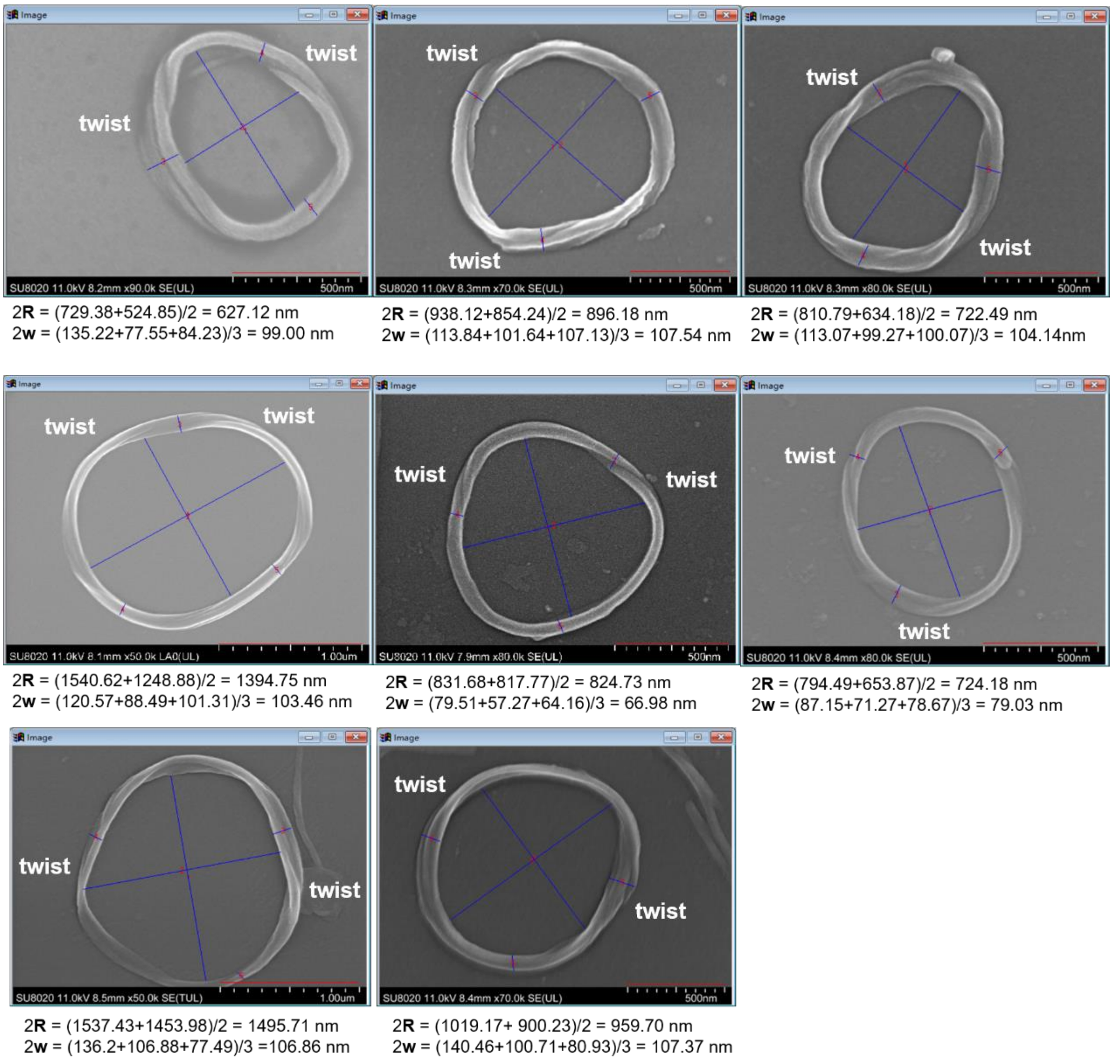

Supplementary Figure 10. SEM images of L-**3** self-assemblies showed chiral toroidal structures with two twists, [L-**3**] = 11 mM, pH =2. 2**R** = (**a**+**b**)/2, **a**: long diameter, **b**: short diameter, 2**w**: average fiber wideness. Scar bars are marked at the bottom right corner.

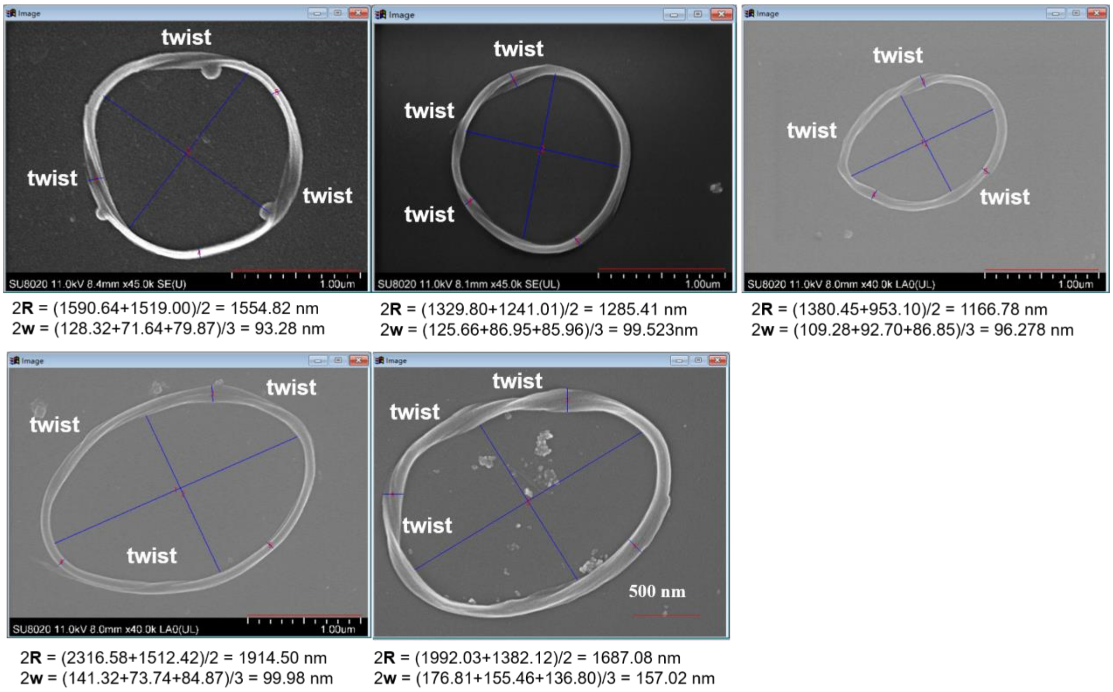

Supplementary Figure 11. SEM images of L-**3** self-assemblies showed chiral toroidal structures with three twists, [L-**3**] = 11 mM, pH =2. 2**R** = (**a**+**b**)/2, **a**: long diameter, **b**: short diameter, 2**w**: average fiber wideness. Scar bars are marked at the bottom right corner.

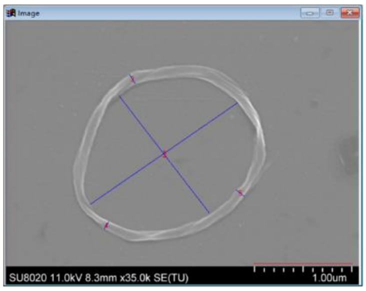

2R = (1815.84 + 1504.59)/2 = 1660.22 nm
2w = (120.72+91.00+106.07)/3 = 105.93 nm

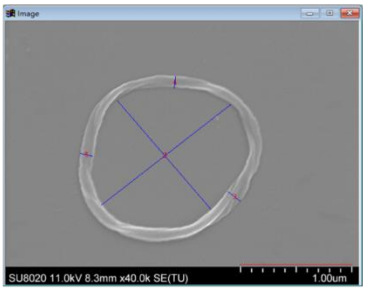

2R = (1467.43+1286.18)/2 = 1376.81 nm
2w = (145.69+110.69+118.13)/3 = 124.84 nm

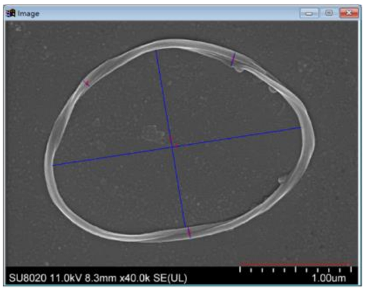

2R = (2262.97+1609.37)/2 = 1936.17 nm
2w = (126.79+78.32+97.25)/3 = 100.79 nm

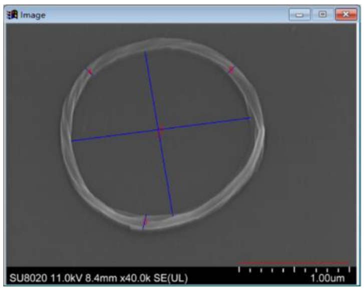

2R = (1657.50+1537.91)/2 = 1597.71 nm
2w = (141.35+106.20+97.35)/3 = 114.97 nm

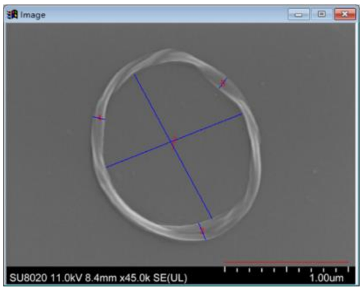

2R = (1325.39+1175.48)/2 = 1250.44 nm
2w = (154.61+106.11+109.47)/3 = 123.40 nm

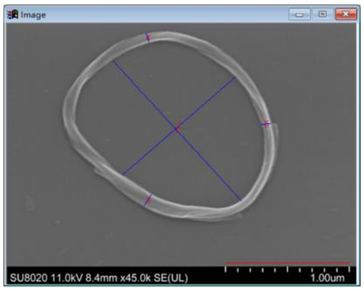

2R = (1538.23+1216.54)/2 = 1377.39 nm
2w = (111.46+87.08+82.60)/3 = 93.71 nm

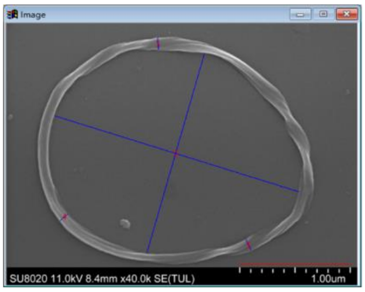

2R = (2294.13+1862.60)/2 = 2078.37 nm
2w = (110.88+91.31+109.97)/3 = 104.05nm

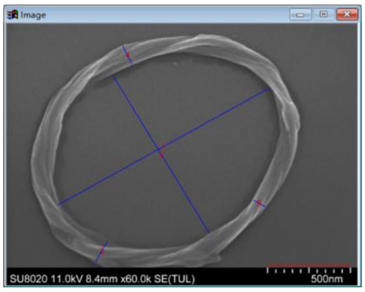

2R = (1448.25+1102.09)/2 = 1275.17 nm
2w = (147.20+80.89+123.07)/3 = 117.05 nm

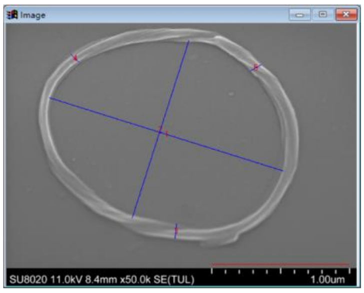

2R = (1787.98+1356.28)/2= 1572.13 nm
2w = (110.19+77.49+98.19)/3 = 95.29 nm

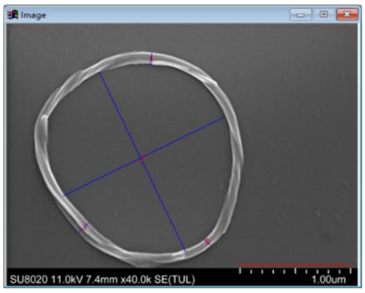

2R = (1787.32+1605.89)/2 = 1696.61 nm
2w = (130.31+111.39+78.65)/3 =106.78 nm

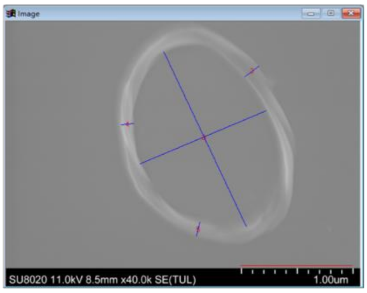

2R = (1726.47+1222.28)/2 = 1474.38 nm
2w = (165.40+116.27+130.47)/3 = 137.38 nm

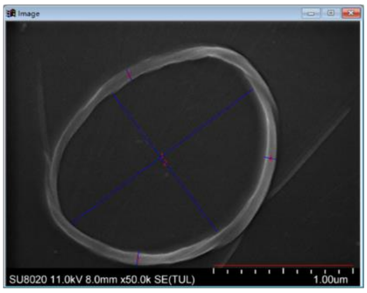

2R = (1627.47 +1242.27)/2 = 1434.87 nm
2w = (107.47+90.21+90.75)/3 = 96.14 nm

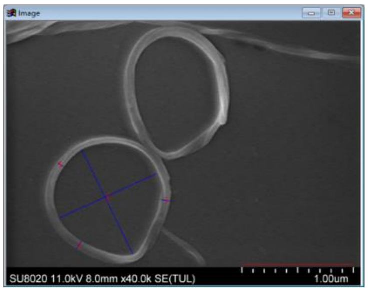

2R = (1044.62+954.29)/2 = 999.46 nm
2w = (90.86+87.73+61.42)/3 =80.00 nm

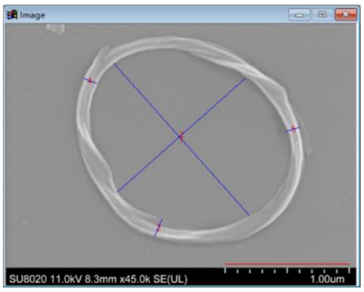

2R = (1635.58 +1375.68)/2 = 1505.63 nm
2w = (154.61+110.68+108.91)/3 =124.73 nm

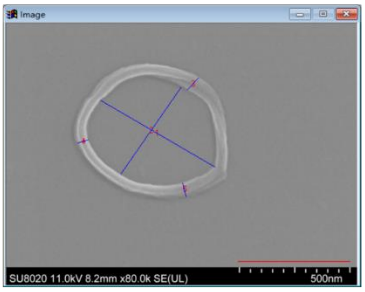

2R = (593.36 +471.89)/2 = 532.63 nm
2w = (75.59+51.12+63.01)/3 =63.24 nm

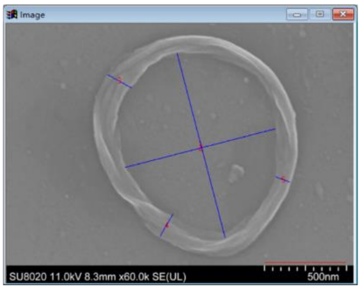

2R = (1174.14+955.16)/2 = 1064.65 nm
2w = (171.70+158.97+93.67)/3 = 141.45 nm

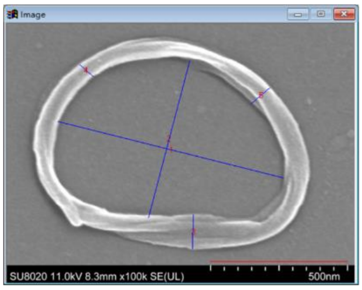

2R = (845.42+592.28)/2 = 718.85 nm
2w = (125.97+69.83+94.41)/3 = 96.74 nm

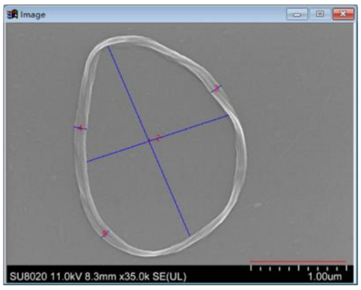

2R = (2152.57+1557.05)/2 = 1854.81 nm
2w = (136.85+135.47+124.74)/3 = 132.35 nm

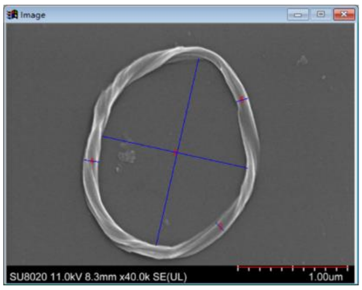

2R = (1719.97+1333.96)/2 = 1526.97 nm
2w = (111.57+134.57+107.42)/3 = 117.85 nm

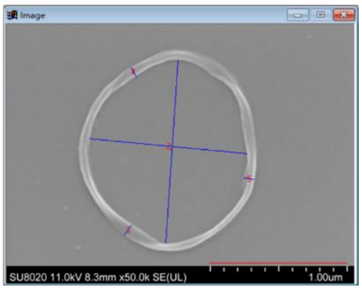

2R = (1331.50+1148.01)/2 = 1239.76 nm
2w = (94.78+86.34+79.44)/3 = 86.85 nm

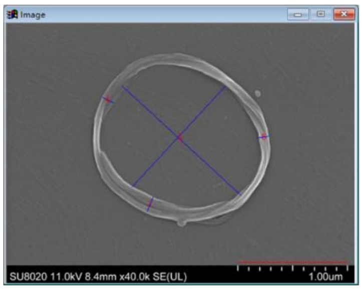

2R = (1487.52+1263.18)/2 = 1375.35 nm
2w = (147.97+116.75+107.23)/3 = 123.98 nm

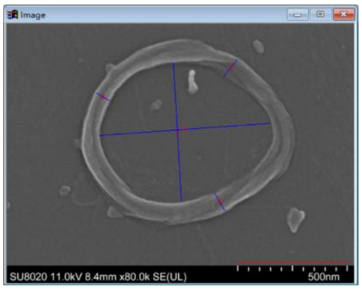

2R = (770.26+627.68)/2 = 698.97 nm
2w = (102.05+71.16+91.69)/3 = 88.30 nm

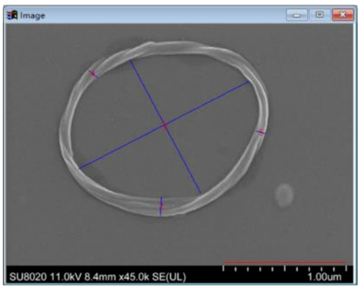

2R = (1563.73+1233.90)/2 = 1398.82 nm
2w = (148.84+97.92+85.31)/3 = 110.69 nm

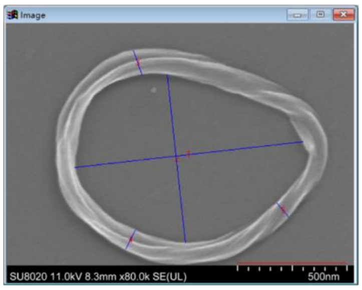

2R = (1053.25 +779.81)/2 = 916.53 nm
2w = (122.07+97.42+86.02)/3 = 101.84 nm

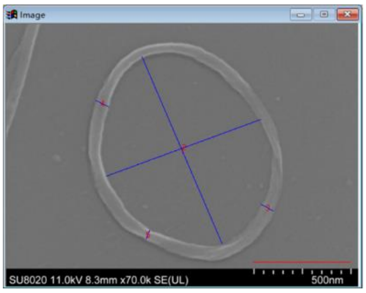

2R = (1048.11+848.00)/2 = 948.06 nm
2w = (72.55+74.06+49.15)/3 = 65.25 nm

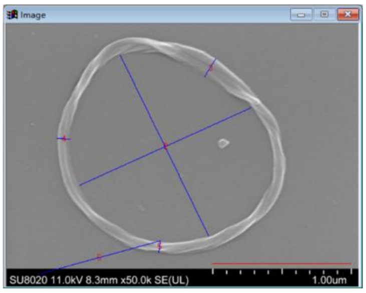

2R = (1457.65 +1358.77)/2 = 1408.21 nm
2w = (158.24+99.13+89.89)/3 = 115.75 nm

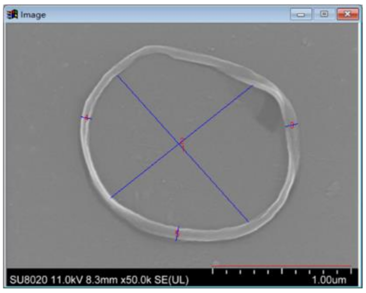

2R = (1418.32+1309.18)/2 = 1363.75 nm
2w = (90.84+80.20+105.57)/3 = 92.20 nm

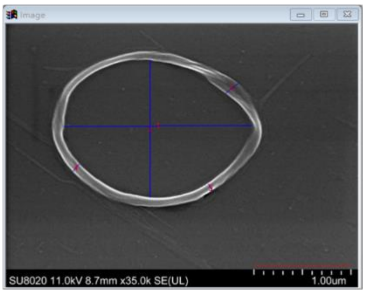

2R = (971.64+711.05)/2 = 841.35 nm
2w = (85.93+52.79+41.43)/3 = 60.05 nm

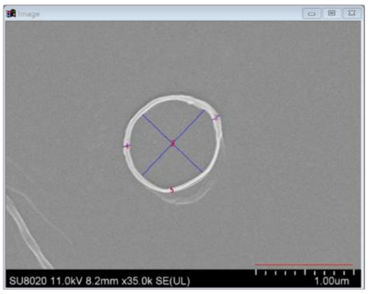

2R = (2435.19+2112.08)/2 = 2273.64 nm
2w = (258.69+148.95+190.06)/3 = 199.23 nm

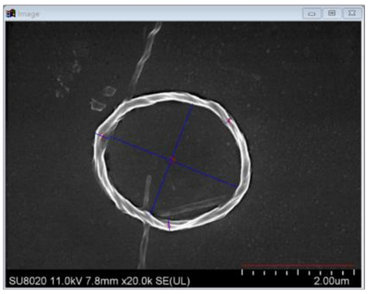

2R = (922.70+858.18)/2 = 890.44 nm
2w = (92.57+79.72+40.73)/3 = 71.01 nm

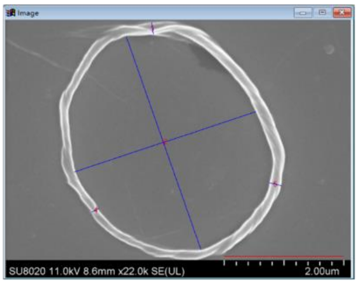

2**R** = (3787.40+3202.51)/2 = 3494.96 nm
2**w** = (204.41+142.04+216.96)/3 = 187.80 nm

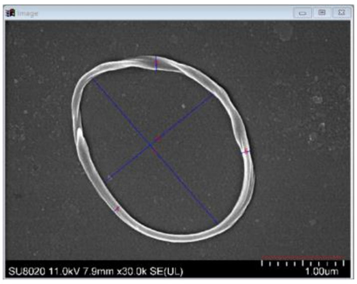

2**R** = (2380.09+1723.86)/2 = 2051.98 nm
2**w** = (183.64+104.79+123.09)/3 = 103.84 nm

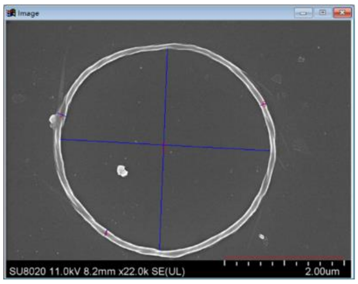

2**R** = (3498.08+3378.52)/2 = 3438.30 nm
2**w** = (154.64+112.23+112.04)/3 = 126.30 nm

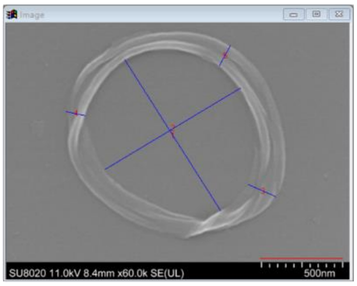

2**R** = (1094.79+963.42)/2 = 1029.11 nm
2**w** = (182.10+120.40+144.01)/3 = 148.84 nm

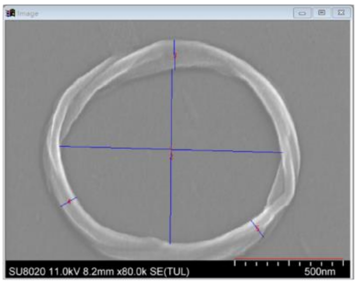

2**R** = (1029.51+808.70)/2 = 919.11 nm
2**w** = (140.55+88.98+98.56)/3 =109.36 nm

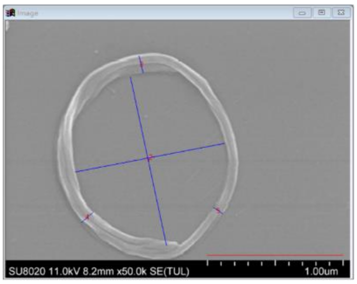

2**R** = (1271.34+1121.38)/2 = 1196.36 nm
2**w** = (130.25+119.61+81.99)/3 =110.62 nm

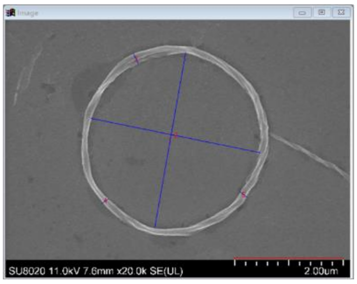

2**R** = (3250.33+3162.79)/2 = 3206.56 nm
2**w** = (201.77+151.12+180.29)/3 = 177.73 nm

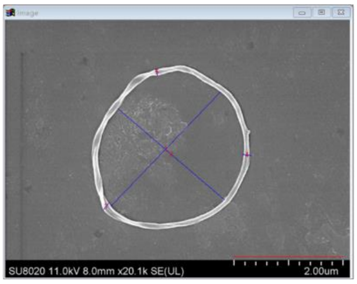

2**R** = (2876.29+2538.09)/2 = 2707.19 nm
2**w** = (176.69+156.82+116.10)/3 = 149.87 nm

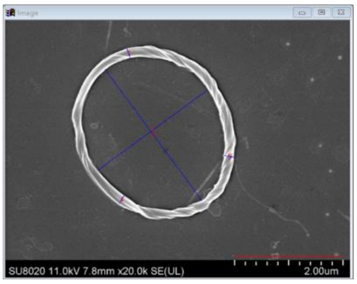

2**R** = (3032.08+2521.25)/2 = 2776.67 nm
2**w** = (192.16+177.34+183.66)/3 = 184.59 nm

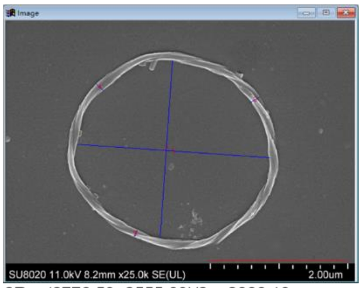

2**R** = (2776.59+2555.60)/2 = 2666.10 nm
2**w** = (165.14+160.04+101.30)/3 = 142.16 nm

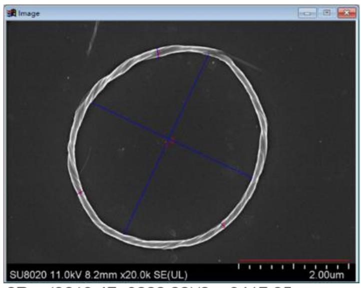

2**R** = (3613.47+3222.22)/2 = 3417.85 nm
2**w** = (192.50+127.24+134.12)/3 = 151.29 nm

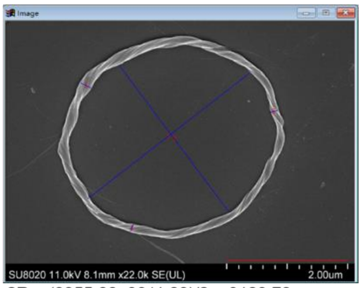

2**R** = (3355.66+3011.89)/2 = 3183.78 nm
2**w** = (191.75+132.90+147.87)/3 = 157.51 nm

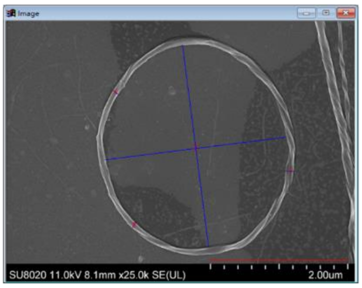

2**R** = (1473.39+1317.00)/2 = 1395.20 nm
2**w** = (63.17+53.85+47.97)/3 = 55.00 nm

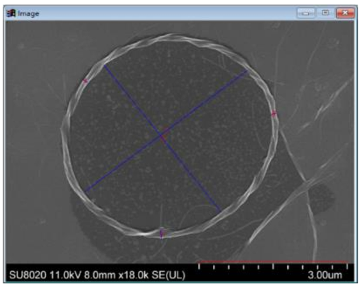

2**R** = (4043.98+3756.83)/2 = 3900.41 nm
2**w** = (173.84+108.92+124.31)/3 = 135.69 nm

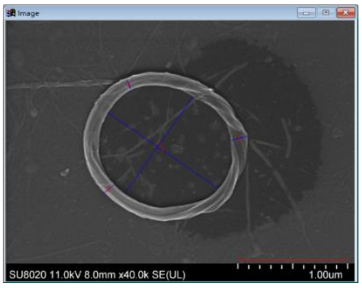

2**R** = (1219.85+1077.81)/2 = 1148.83 nm
2**w** = (146.82+108.71+112.00)/3 = 122.51 nm

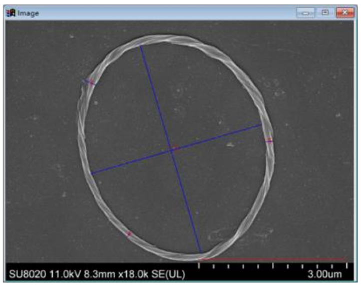

2R = (4375.11+3595.50)/2 = 3985.31 nm
2w = (241.68+132.22+152.20)/3 = 185.37 nm

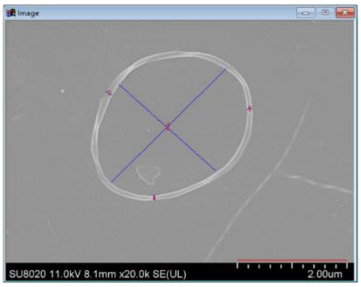

2R = (2966.36+2385.61)/2 = 2675.99 nm
2w = (147.09+108.15+107.92)/3 = 121.05 nm

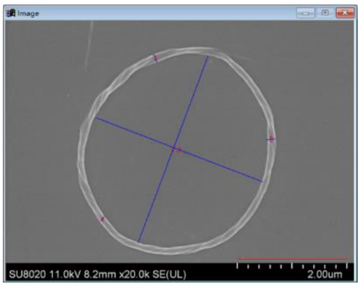

2R = (3637.77+3259.70)/2 = 3448.74 nm
2w = (160.63+119.23+158.25)/3 = 146.04 nm

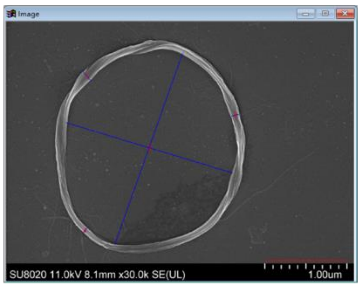

2R = (2453.24+2104.26)/2 = 2278.75 nm
2w = (148.86+95.25+101.84)/3 = 115.32 nm

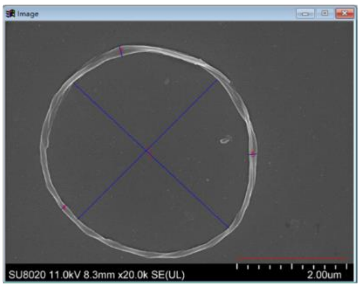

2R = (3836.40+3568.94)/2 = 3702.67 nm
2w = (182.30+155.01+146.21)/3 = 161.17 nm

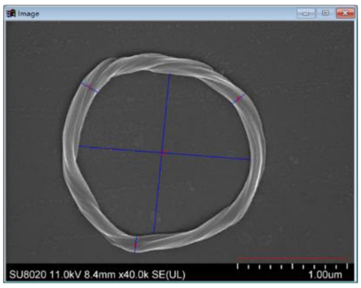

2R = (1570.09+1455.10)/2 = 1512.60 nm
2w = (176.10+147.76+153.04)/3 = 158.97 nm

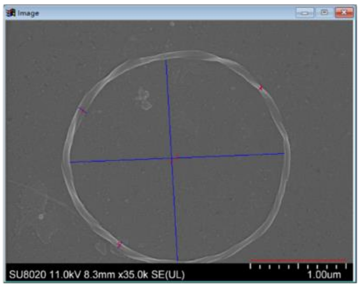

2R = (4138.59+3929.76)/2 = 4034.18 nm
2w = (233.36+184.13+149.13)/3 = 188.87 nm

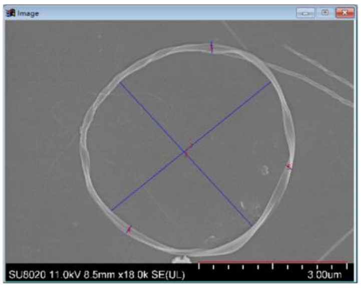

2R = (2250.33+2105.09)/2 = 2177.71 nm
2w = (104.35+42.90+82.55)/3 = 76.60 nm

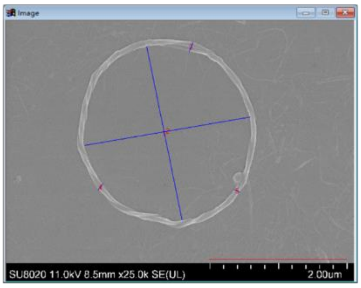

2R = (2559.05+2428.33)/2 = 2493.69 nm
2w = (154.14+130.89+93.89)/3 = 126.31 nm

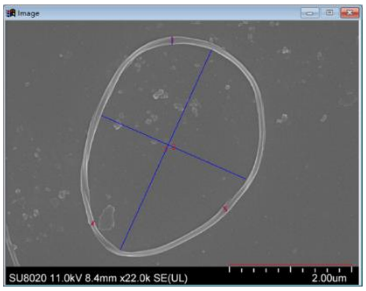

2R = (3594.97+2571.86)/2 = 3083.42 nm
2w = (124.67+94.84+101.85)/3 = 107.12 nm

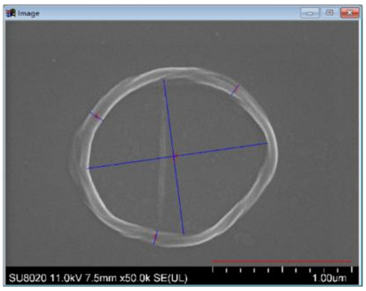

2R = (1302.56+1116.66)/2 = 1209.61 nm
2w = (93.43+118.99+104.16)/3 = 105.53 nm

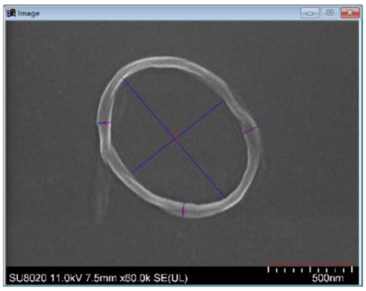

2R = (950.85+ 697.94)/2 = 824.40 nm
2w = (104.82+80.50+91.71)/3 = 92.34 nm

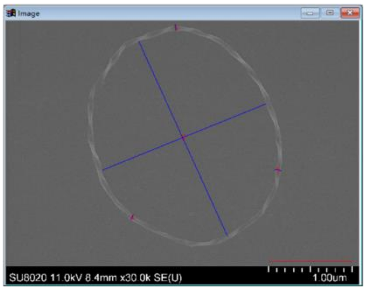

2R = (2573.19+2120.48)/2 = 2346.84 nm
2w = (70.93+72.63+78.74)/3 = 74.10 nm

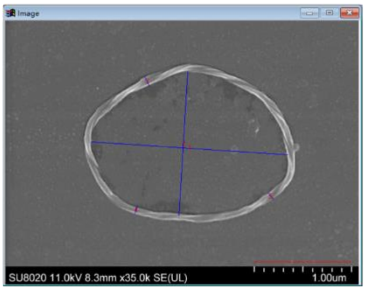

2R = (2016.43+1487.94)/2 = 1752.19 nm
2w = (107.95+82.10+85.73)/3 = 91.93 nm

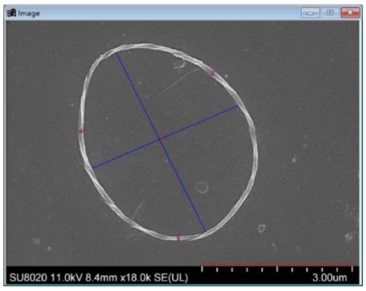

2R = (4005.87+3177.07)/2 = 3591.47 nm
2w = (116.14+119.52+93.96)/3 = 109.87 nm

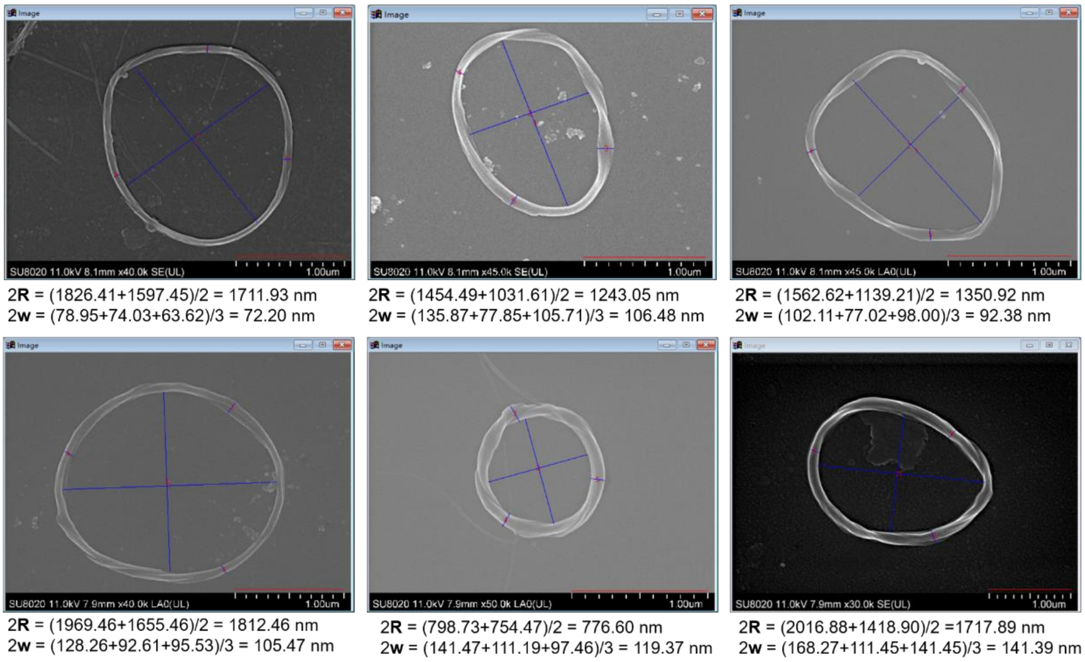

Supplementary Figure 12. SEM images of L-**3** self-assemblies showed chiral toroidal structures (total number is 66) with varied twists, [L-**3**] = 11 mM, pH =2. 2**R** = (**a**+**b**)/2, **a**: long diameter, **b**: short diameter, 2**w**: average fiber wideness. Scar bars are marked at the bottom right corner.

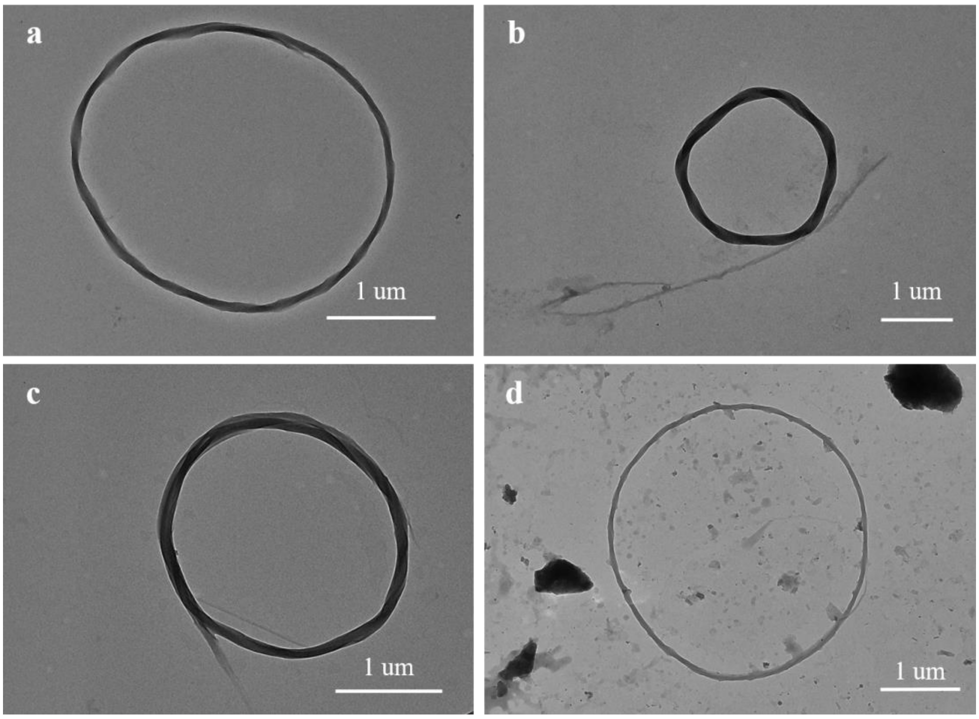

Supplementary Figure 13. TEM images of L-**3** self-assemblies showed toroidal structures. [L-**3**] = 11 mM, pH =2.

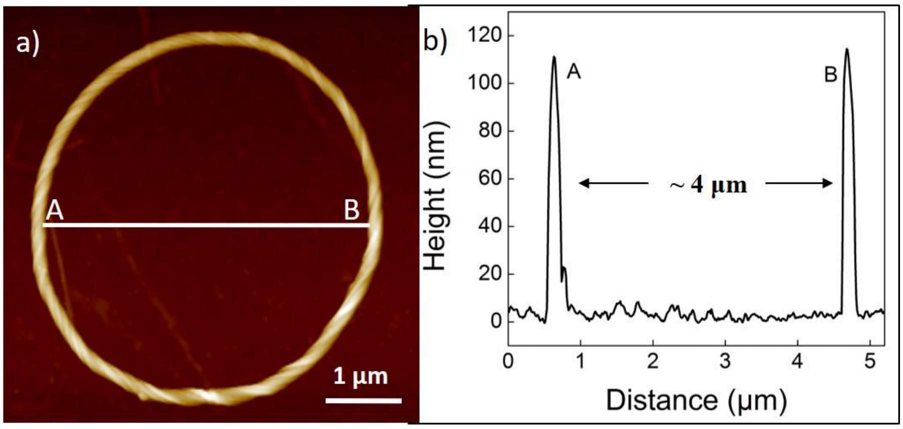

Supplementary Figure 14. **a**, AFM image of chiral nano-toroid formed from D-**3** amphiphile; **b**, AFM height profile along the line in the image **a**. [D-**3**] = 11 mM, pH =2.

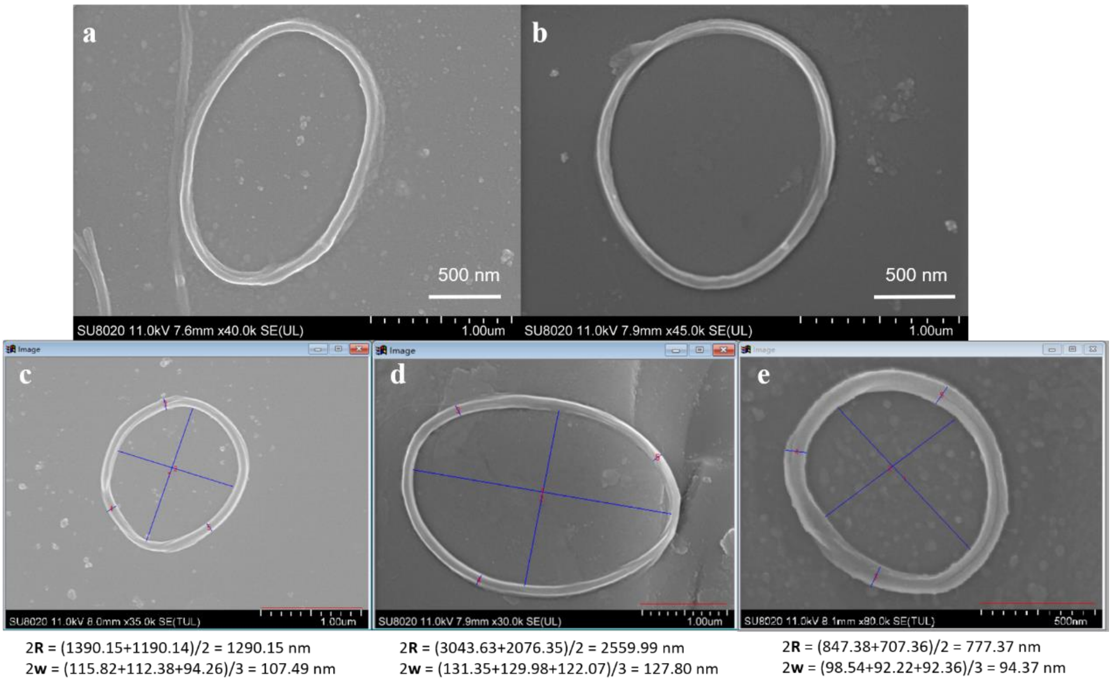

Supplementary Figure 15. SEM images of self-assemblies of racemate L-**3**/D-**3** (molar ratio 1:1) showed achiral toroidal fibers, total concentration is 0.11 mM, pH =2. Scar bars are marked at the bottom right corner.

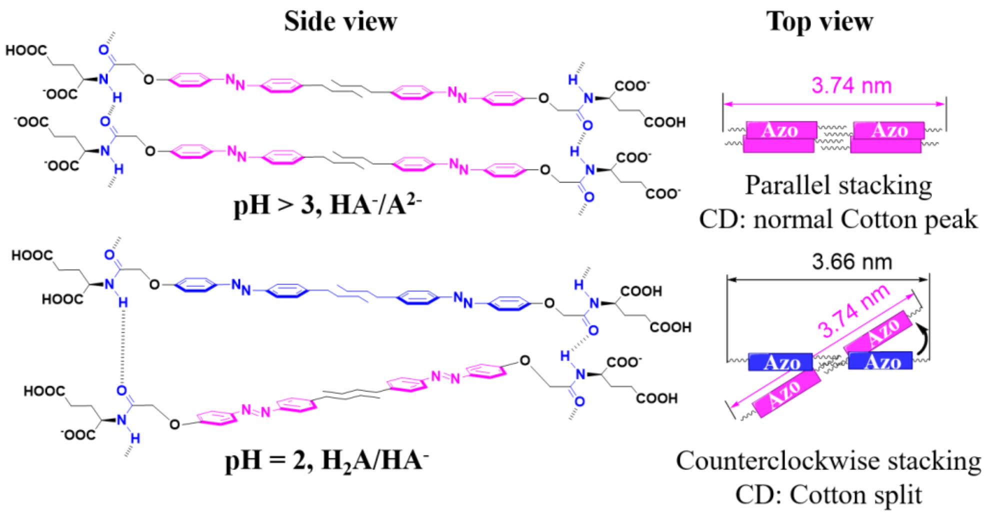

Supplementary Figure 16. Proposed stacking mode of L-**3** amphiphile at different pH values.

a

$$x = \left[R + s * \cos\left(\frac{3}{2}t\right)\right]\cos t$$

$$y = \left[R + s * \cos\left(\frac{3}{2}t\right)\right]\sin t$$

$$z = s * \sin\left(\frac{3}{2}t\right)$$

$$\text{for } s \in [-w, w],\ t \in [0, 2\pi)$$

b

$$x = \left[R + s * \cos\left(\frac{5}{2}t\right)\right]\cos t$$

$$y = \left[R + s * \cos\left(\frac{5}{2}t\right)\right]\sin t$$

$$z = s * \sin\left(\frac{5}{2}t\right)$$

$$\text{for } s \in [-w, w],\ t \in [0, 2\pi)$$

Supplementary Figure 17. Mathematic equations for multiple twisted Möbius strip. **a**, Triply twisted Möbius strip. **b**, Quintuple twisted Möbius strip.

For triply twisted Möbius Strip, its mathematic equations are showed in **Supplementary Figure 17a**, according to our MD calculation, the calculation ratio of R/w is about 8.9. The corresponding theoretical triply twisted Möbius strip image is shown in Figure **6e**. We then measured the triply twisted Möbius strip observed on SEM, as showed in Figure **6e**, its parameters are as follows: the average width of strip is about 2w = 200 nm, and the average midcircle of radius R is 912.5 nm ($R = (R_1 + R_2)/2$, R_1 = 1075 nm, R_2 = 750 nm), so the experiment ratio R/w for triply twisted Möbius strip is 9.13.

For quintuple twisted Möbius strip observed on SEM, its parameters are as follows: the average width of strip is about 2w = 240 nm, and the average midcircle of radius

R is 1125 nm ($R = (R_1 + R_2)/2$, R_1 = 1250 nm, R_2 = 1000 nm), so the experiment ratio R/w for triply twisted Möbius strip is 9.37.

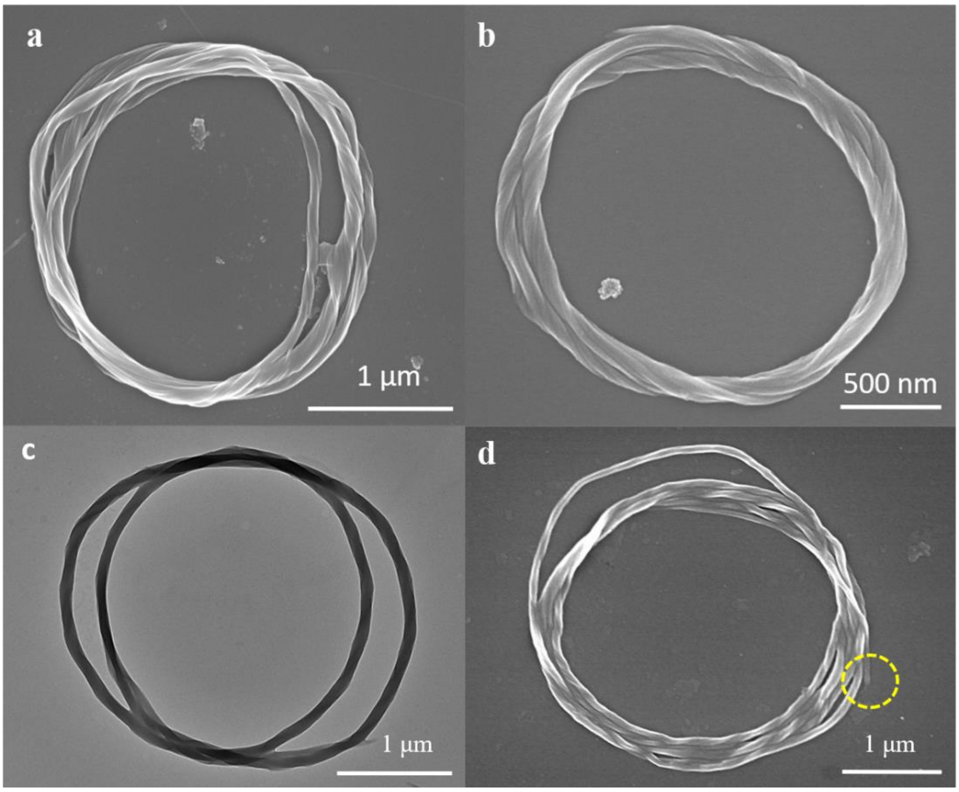

Supplementary Figure 18. Special nano-structures in the self-assemblies of L-**3** and D-**3** (pH 2.0). **a**, Left-handed closed concentric disc structures from L-**3** assemblies; **b**, Right-handed catenane-like nano structure from D-**3** assemblies; **c**, TEM image showed a catenane-like nano structure from L-**3** assemblies; d, Uncyclized concentric disc structures from L-**3** assemblies, the yellow dashed cycle indicates the open end.

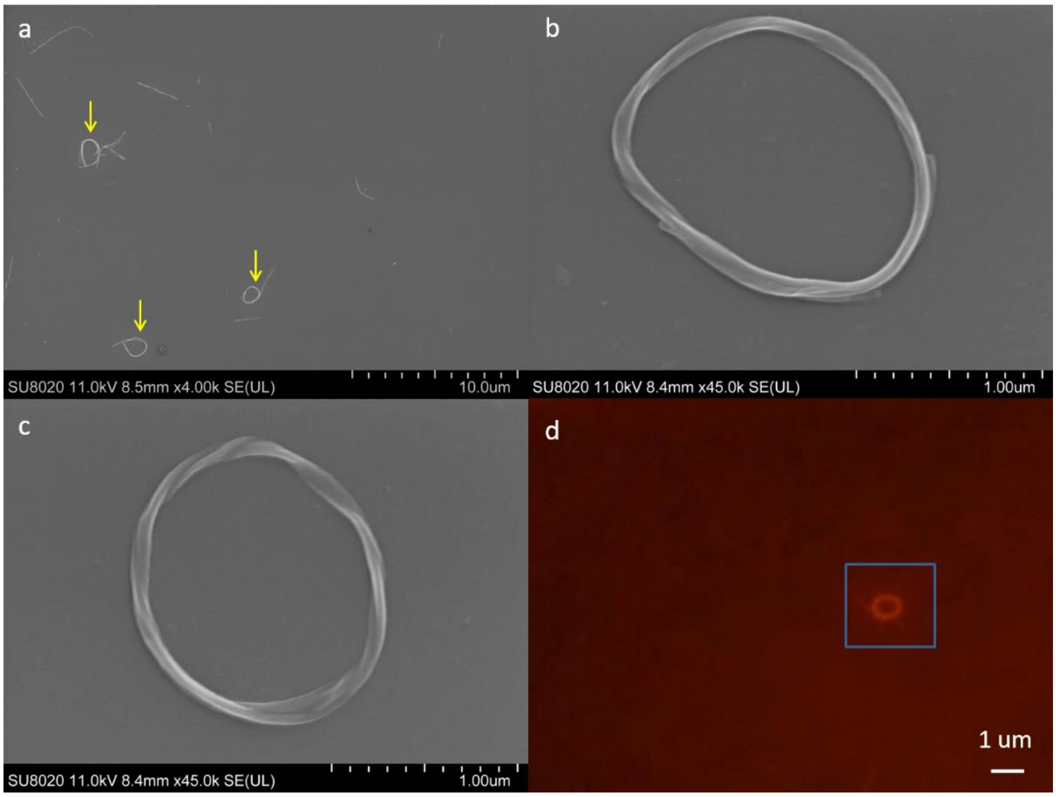

Supplementary Figure 19. SEM and fluorescence microscope images of assemblies of D-3 doped with the organic dye rhodamine B (RhB). The concentration of D-**3** is 0.11 mM in water, pH = 2, the ratio of RhB/D-**3** is 1/10. **a-c**, SEM images. **d**, Fluorescence microscope image. Due to the relatively lower magnification, the helicity of nano-toroid by fluorescence microscopy was not distinguishable. However, the chirality of nano-toroids are remained according to SEM observations.

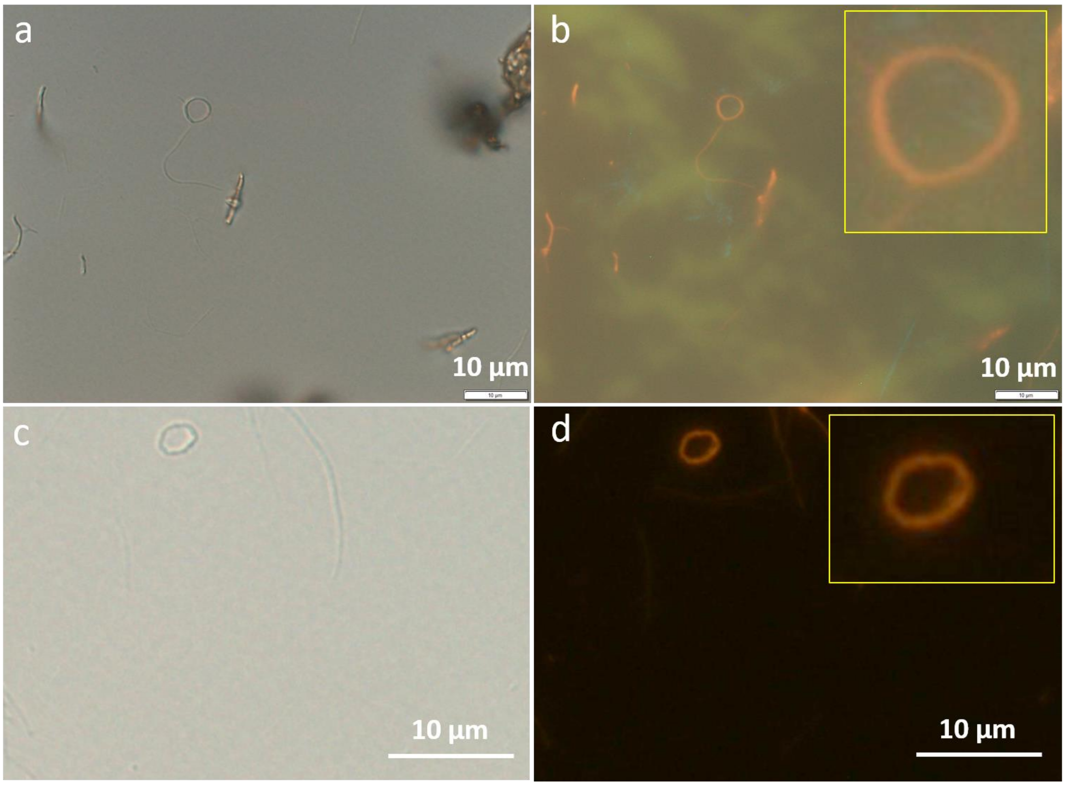

Supplementary Figure 20. Fluorescence microscope images of assemblies of D-3 doped with the organic dyes thioflavin T (ThT) and acridine orange (AO). The concentration of D-**3** is 0.11 mM in water, pH = 2, the ratio of organic dyes/D-**3** is 1/10. **a-b**, Optical microscope and fluorescence microscope image of D-**3**/ThT. **c-d**. Optical microscope and fluorescence microscope image of D-**3**/AO.

2. Synthesis and characterization of glutamic amphiphiles

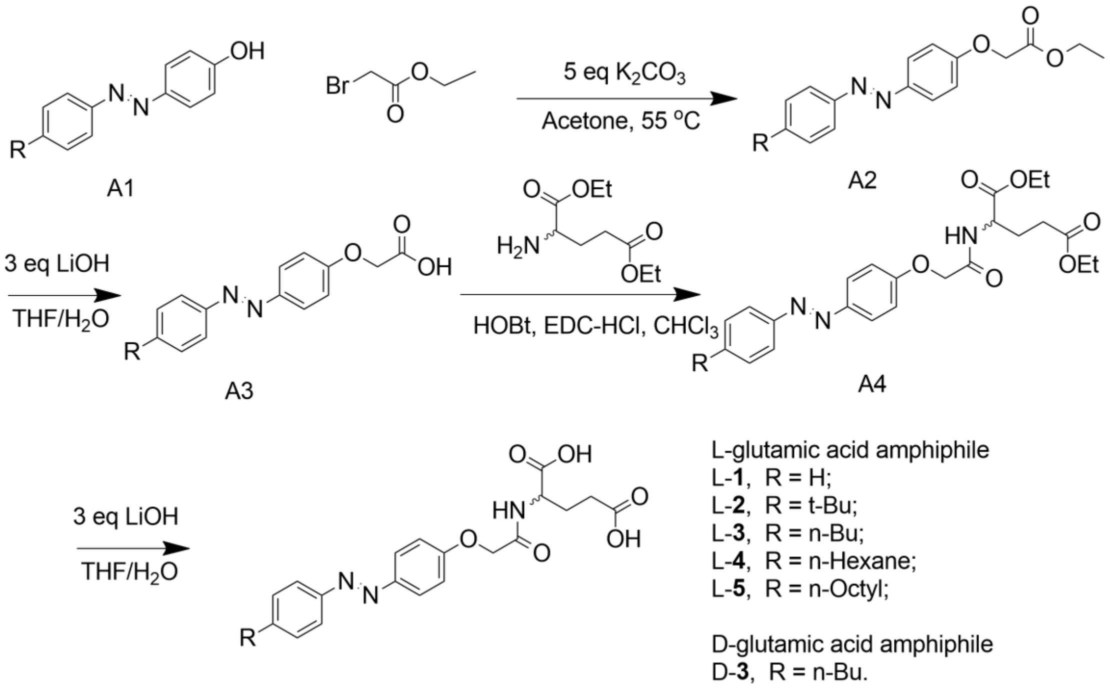

Supplementary Figure 21. The synthesis routes of amphiphiles L-**1**-L-**5** and D-**3**.

All the starting materials were purchased from TCI company and used as received without further purification. All the amphiphiles are synthesized through similar methods. The detailed synthesis method of representative compound L-**3** is as following:

Compound L-3: Starting material compound A1 (2.54 g, 10.00 mmol), ethyl 2-bromoacetate (1.83 g, 11.00 mmol) were dissolved in 100 mL acetone, then K_2CO_3 (6.90 g, 50.00 mmol) was added to the above solution, heat to 55 ℃, and the resulting mixture was stirred at this temperature overnight. After cooling to room temperature, the mixture was filtered by filter paper and the solvent was removed under reduced pressure to give a crude product. This crude product was dissolved in mixed solution (THF/H_2O = 3:1, 50.00 mL), LiOH (1.20 g, 50.00 mmol) was added and then stirred at room temperature for 6 h. Adding hydrochloric acid solution to the above reaction mixture to adjust pH to 2.0. The reaction mixture was extracted with $CHCl_3$ for 3 times (50 mL × 3). The organic phase was collected and dried with anhydrous Na_2SO_4 (10.00 g). After being filtered, the organic solvent was removed using rotary evaporator and the obtained crude product was purified by recrystallization, 2.65 g product of A3 was obtained. Then L-Glutamic acid diethyl ester hydrochloride (2.18 g, 11.00 mmol) was dissolved in $CHCl_3$, and triethylamine (1.80 g, 11.00 mmol) was added to the above solution, A3 (2.65 g, 8.52 mmol) and 1-Hydroxybenzotriazole (HOBt) (1.33 g, 8.92 mmol) and 1-(3-Dimethylaminopropyl)-3-ethylcarbodiimide hydrochloride (EDC-HCl) (1.87 g, 8.92 mmol) were added to the above solution, the mixed solution was stirred at room

temperature overnight. After monitored by TLC, saturated Na_2CO_3 aqueous solution was added to the above reaction solution to remove water soluble by-products, then the organic phase was collected and dried with anhydrous Na_2SO_4. After filtration, the organic solvent was removed using rotary evaporator, giving crude product A4 (3.53 g, 7.20 mmol), which was purified by recrystallization. The obtained product A4 was dissolved in mixed solution (THF/H_2O = 3:1, 50 mL), LiOH (0.85 g, 36.00 mmol) was added and stirred at room temperature for 6 h. Adding hydrochloric acid aqueous solution to adjust pH to 2. The reaction mixture was extracted with $CHCl_3$ for 3 times (50 mL × 3). Finally, the organic phase was collected and dried with anhydrous Na_2SO_4, after being filtered, the organic solvent was removed using rotary evaporator and the obtained crude product was purified by fast column chromatography and recrystallization. The product of L3 (2.87 g, 6.50 mmol) was a yellow solid, yield 65.0 %. ^1H NMR (400 MHz, D_6-DMSO, 298K, δ, ppm)：0.90-0.93 (t, 3H, CH_3), 1.31-1.37 (m, 2H, CH_2), 1.57-1.62 (m, 2H, CH_2), 1.84-1.92 (m, 1H, CH_2), 1.99-2.06 (m, 1H, CH_2), 2.27-2.31 (t, 2H, CH_2), 2.65-2.69 (t, 2H, CH_2), 4.28-4.34 (m, 1H, CH), 4.67-4.68 (d, 2H, CH_2), 7.14-7.16 (d, 2H, Ar-H), 7.38-7.40 (d, 2H, Ar-H), 7.76-7.79 (d, 2H, Ar-H), 7.85-7.88 (d, 2H, Ar-H), 8.40-8.41 (d, 1H, NH). ^{13}C NMR (D_4-CD_3OD, 100 MHz, 298 K): 14.25, 23.35, 27.58, 31.15, 34.69, 36.45, 52.89, 68.22, 116.32, 123.62, 125.52, 130.14, 147.42, 148.95, 152.24, 161.35, 170.80, 174.45, 176.41. MALDI-TOF MS: calcd. for $C_{23}H_{27}N_3O_6$ M^+: m/z 441.4; found $[M+Na]^+$: m/z = 464.2.

Other amphiphile derivatives L-**1**, L-**2**, L-**4**, L-**5** and D-**3** were synthesized as the above steps of L-**3**.

Compound L-1: ^1H NMR (400 MHz, D_6-DMSO, 298K, δ, ppm)：1.82-1.92 (m, 1H, CH_2), 2.00-2.09 (m, 1H, CH_2), 2.28-2.31 (m, 2H, CH_2), 3.32 (s, 2H, CH_2), 4.29-4.35 (m, 1H, CH), 4.68-4.69 (d, 2H, CH_2), 7.15-7.17 (d, 2H, Ar-H), 7.51-7.60 (m, 1H, Ar-H), 7.84-7.86 (d, 2H, Ar-H), 7.89-7.91 (d, 2H, Ar-H), 8.42-8.44 (d, 1H, N-H). MALDI-TOF MS: calcd. for $C_{19}H_{19}N_3O_6$ M^+: m/z 385.4; found $[M+Na]^+$: m/z = 408.2.

Compound L-2: ^1H NMR (400 MHz, D_6-DMSO, 298K, δ, ppm)：1.34 (s, 9H, CH_3), 1.83-1.92 (m, 1H, CH_2), 2.00-2.09 (m, 1H, CH_2), 2.28-2.31 (m, 2H, CH_2), 3.32 (m, 2H, CH_2), 4.29-4.35 (m, 1H, CH), 4.67-4.68 (d, 2H, CH_2), 7.14-7.16 (d, 2H, Ar-H), 7.58-7.61 (d, 2H, Ar-H), 7.78-7.81 (d, 2H, Ar-H), 7.86-7.88 (d, 2H, Ar-H), 8.40-8.42 (d, 1H, N-H). MALDI-TOF MS: calcd. for $C_{23}H_{27}N_3O_6$ M^+: m/z 441.4; found $[M+Na]^+$: m/z = 464.2.

Compound L-4: ^1H NMR (400 MHz, D_6-DMSO, 298K, δ, ppm)：0.84-0.88 (t, 3H, CH_3), 1.29 (m, 6H, CH_2), 1.57-1.63 (t, 2H, CH_2), 1.82-1.92 (m, 1H, CH_2), 2.01-2.09 (m, 1H, CH_2), 2.25-2.31 (m, 2H, CH_2), 2.64-2.68 (t, 2H, CH_2), 4.29-4.35 (m, 1H, CH), 4.67-4.68 (d, 2H, CH_2), 7.14-7.16 (d, 2H, Ar-H), 7.38-7.40 (d, 2H, Ar-H), 7.76-7.78

(d, 2H, Ar-H), 7.86-7.88 (d, 2H, Ar-H), 8.42-8.44 (d, 1H, N-H). MALDI-TOF MS: calcd. for $C_{25}H_{31}N_3O_6$ M^+: m/z 469.5; found $[M+Na]^+$: m/z = 492.3.

Compound L-5: ^{1}H NMR (400 MHz, D_6-DMSO, 298K, δ, ppm): 0.83-0.87 (t, 3H, CH_3), 1.25-1.30 (m, 12H, CH_2), 1.59-1.63 (t, 2H, CH_2), 1.83-1.92 (m, 1H, CH_2), 2.01-2.10 (m, 1H, CH_2), 2.28-2.32 (m, 2H, CH_2), 2.64-2.68 (t, 2H, CH_2), 4.30-4.35 (m, 1H, CH), 4.67-4.68 (d, 2H, CH_2), 7.14-7.16 (d, 2H, Ar-H), 7.37-7.39 (d, 2H, Ar-H), 7.76-7.78 (d, 2H, Ar-H), 8.42-8.44 (d, 1H, N-H). MALDI-TOF MS: calcd. for $C_{27}H_{35}N_3O_6$ M^+: m/z 497.6; found $[M+Na]^+$: m/z = 520.3.
